# Characterization of Profilin Polymorphism in Pollen with a Focus on Multifunctionality

**DOI:** 10.1371/journal.pone.0030878

**Published:** 2012-02-14

**Authors:** Jose C. Jimenez-Lopez, Sonia Morales, Antonio J. Castro, Dieter Volkmann, María I. Rodríguez-García, Juan de D. Alché

**Affiliations:** 1 Department of Biochemistry, Cell and Molecular Biology of Plants, Estación Experimental del Zaidín, High Council for Scientific Research, Granada, Spain; 2 Institute of Cellular and Molecular Botany, Department of Plant Cell Biology, University of Bonn, Bonn, Germany; University of South Florida College of Medicine, United States of America

## Abstract

Profilin, a multigene family involved in actin dynamics, is a multiple partners-interacting protein, as regard of the presence of at least of three binding domains encompassing actin, phosphoinositide lipids, and poly-L-proline interacting patches. In addition, pollen profilins are important allergens in several species like *Olea europaea* L. (Ole e 2), *Betula pendula* (Bet v 2), *Phleum pratense* (Phl p 12), *Zea mays* (Zea m 12) and *Corylus avellana* (Cor a 2). In spite of the biological and clinical importance of these molecules, variability in pollen profilin sequences has been poorly pointed out up until now. In this work, a relatively high number of pollen profilin sequences have been cloned, with the aim of carrying out an extensive characterization of their polymorphism among 24 olive cultivars and the above mentioned plant species. Our results indicate a high level of variability in the sequences analyzed. Quantitative intra-specific/varietal polymorphism was higher in comparison to inter-specific/cultivars comparisons. Multi-optional posttranslational modifications, e.g. phosphorylation sites, physicochemical properties, and partners-interacting functional residues have been shown to be affected by profilin polymorphism. As a result of this variability, profilins yielded a clear taxonomic separation between the five plant species. Profilin family multifunctionality might be inferred by natural variation through profilin isovariants generated among olive germplasm, as a result of polymorphism. The high variability might result in both differential profilin properties and differences in the regulation of the interaction with natural partners, affecting the mechanisms underlying the transmission of signals throughout signaling pathways in response to different stress environments. Moreover, elucidating the effect of profilin polymorphism in adaptive responses like actin dynamics, and cellular behavior, represents an exciting research goal for the future.

## Introduction

Plant cytoskeleton plays a major role during cell events including division, expansion, morphogenesis and differentiation, and also in response to external stimuli like pathogen attack [1]. Actin, the central player in the cytoskeleton, has a paramount importance in the cytoskeleton structure. Its proper assembly and organization depends upon the expression of an appropriate and complex mixture of actin-binding proteins (ABPs) as well as signals mediated by various signaling pathways involving molecules like the Rho family of GTPases [2]. Plant cells respond to a wide range of internal or external stimuli by reorganizing their cytoplasm [3]. These modifications often correlate with changes in the actin filament network, being the ABPs at the crossroad between extracellular signals and rearrangements of the cytoskeleton. One of the best characterized examples of ABPs in plants is profilin [4], a large multigene family (Pfam accession number PF00235) [5], differentially expressed, with biochemical and functionally diverse isoforms [6]. They have been also found in lower eukaryotes [7], invertebrates [8], and vertebrates [9]. Viral profilins have been found, whose gene organization is homologous to mammalian profilins [10].

Plant profilins contain up to ten different genes in both mono- and dicotyledonous [11], divided in two classes differentially expressed: one is ubiquitously present, and constitutively expressed in all plant tissues, whereas the second class is restricted to the reproductive tissues [12]. The complexity of profilin expression and the number of isoforms in higher plants is correlated with the observation that the actin family is also more complex in plants than in other kingdoms [13].

Profilins display a molecular mass around 15 kDa. These proteins control actin polymerization in eukaryotic cells [14], promoting or inhibiting actin polymerization, depending on the profilin/G-actin ratio, ionic environment of the cell [15], and also depending of the interaction with other actin-binding proteins [16]. Profilins have been shown to be in a 1∶1 stoichiometry rate in relation with total actin in pollen [17] and in tobacco suspension cells [18]. They are generally considered to be the main buffer of the actin monomer pool, suppressing filament spontaneous nucleation and providing a large population of subunits for formin-mediated polymerization [19].

Profilins have been revealed as key mediators of the membrane–cytoskeleton communication, acting at critical points of signaling pathways initiated by events in the plasma membrane and transmitted by transduction cascades to promote cytoskeletal rearrangements [20]. This functionality arises from their binding capacity of interaction with phosphatidylinositides (PIP2), as well as with poly-L-proline-rich proteins [21].

Up to date, over 400 profilin sequences from 100 plant species are available at NCBI GenBank database [22]. Among these, about half have been isolated from pollen, mostly from allergenic plant species including the allergens Ole e 2, Bet v 2, Cor a 2, Phl p 12 and Zea m 12. However, the presence of polymorphism has only been poorly pointed out in a low number of sequences in *Z. mays* [23], *T. aestivum* [24], *A. thaliana* [6], *N. tabacum* [25], *O. europaea* [26], *C. dactylon* [27], *P. pratense* [27], *Artemisia vulgaris* [28], and *P. Judaica* [29]. No data about interespecific comparisons, and cultivars sequence variability is available so far.

In the present study, we have cloned a representative number of profilin sequences from olive pollen and from other 4 worldwide distributed allergenic species (*Betula verrucosa*, *Corylus avellana*, *Phleum pratense* and *Zea mays*), to extensively characterize the polymorphism affecting profilin sequences. A comparative analysis of the intra- and inter-species/cultivars variability has been carried out, as well as an extensive bioinformatic analysis of how the polymorphism may affect these sequence motives considered of relevance for profilin functionality. These include sequences differentially affecting physicochemical properties, posttranslational modifications sites, i.e. phosphorilation, and also the interaction properties of ligand-binging partners.

## Results

### Analysis of profilin sequence polymorphism

RT-PCR amplification of total RNA with a set of degenerate primers resulted in 94 raw sequences from 24 olive cultivars, 10 from hazel, 8 from timothy-grass, 7 from maize and 2 identical sequences from birch. Each one of these sequences was individually analyzed by the nucleotide-nucleotide BLAST (blastn) program and ScanProsite software searching for specific profilin motif patterns. Non-redundant nucleotide sequences were deposited in the GenBank™/EMBL Database (Table 1).

**Table 1:** GenBank™/EMBL Database entries.

*Olea europaea*Cultivar/Clone	Accession n°	*Olea europaea*Cultivar/Clone	Accession n°	Species/Clone	Accession n°
Acebuche 1	DQ138355	Lucio 2	DQ138363	*Betula pendula* 1	DQ650633
Acebuche 2	DQ138356	Lucio 3	DQ138365	*Corylus avellana* 1	DQ663543
Acebuche 3	DQ138357	Lucio 4	DQ138364	*Corylus avellana* 2	DQ663544
Arbequina 1	DQ138327	Lucio 5	DQ640908	*Corylus avellana* 3	DQ663545
Arbequina 2	DQ138328	Manzanilla Sevilla 1	DQ117911	*Corylus avellana* 4	DQ663546
Arbequina 3	DQ138329	Manzanilla Sevilla 2	DQ138324	*Corylus avellana* 5	DQ663547
Arbequina 4	DQ138330	Manzanilla Sevilla 3	DQ138325	*Corylus avellana* 6	DQ663548
Bella de España 1	DQ317563	Manzanilla Sevilla 4	DQ138326	*Corylus avellana* 7	DQ663549
Bella de España 2	DQ317564	Morrut 1	DQ317573	*Corylus avellana* 8	DQ663550
Bella de España 3	DQ640909	Morrut 2	DQ317574	*Corylus avellana* 9	DQ663551
Bella de España 4	DQ640910	Morrut 3	DQ317575	*Corylus avellana* 10	DQ663552
Blanqueta 1	DQ138335	Morrut 4	DQ317576	*Phleum pratense* 1	DQ663535
Blanqueta 2	DQ138336	Picual 1	DQ317580	*Phleum pratense* 2	DQ663536
Blanqueta 3	DQ138337	Picual 2	DQ317581	*Phleum pratense* 3	DQ663537
Blanqueta 4	DQ138338	Picual 3	DQ317582	*Phleum pratense* 4	DQ663538
Cornicabra 1	DQ138331	Picual 4	DQ640904	*Phleum pratense* 5	DQ663539
Cornicabra 2	DQ138332	Picual 5	DQ663553	*Phleum pratense* 6	DQ663540
Cornicabra 3	DQ138333	Picual 6	DQ663554	*Phleum pratense* 7	DQ663541
Cornicabra 4	DQ138334	Picual 7	DQ663555	*Phleum pratense* 8	DQ663542
Empeltre 1	DQ138342	Picual 8	DQ663556	*Zea mays* 1	DQ663559
Empeltre 2	DQ138343	Picual 9	DQ663557	*Zea mays* 2	DQ663560
Empeltre 3	DQ138344	Picual 10	DQ663558	*Zea mays* 3	DQ663561
Farga 1	DQ317565	Picudo 1	DQ117907	*Zea mays* 4	DQ663562
Farga 2	DQ317566	Picudo 2	DQ117908	*Zea mays* 5	DQ663563
Farga 3	DQ317567	Picudo 3	DQ117909	*Zea mays* 6	DQ663564
Frantoio 1	DQ317568	Picudo 4	DQ117910	*Zea mays* 7	DQ663565
Frantoio 2	DQ317569	Sevillenca 1	DQ138348		
Galega 1	DQ317570	Sevillenca 2	DQ138349		
Hojiblanca 1	DQ061979	Sevillenca 3	DQ138350		
Hojiblanca 2	DQ061980	Sourani 1	DQ317577		
Hojiblanca 3	DQ061981	Sourani 2	DQ317578		
Hojiblanca 4	DQ061982	Sourani 3	DQ317579		
Leccino 1	DQ138345	Sourani 4	DQ640905		
Leccino 2	DQ138346	Verdial Huévar 1	DQ117902		
Leccino 3	DQ138347	Verdial Huévar 2	DQ117903		
Lechín de Granada 1	DQ317571	Verdial Huévar 3	DQ117904		
Lechín de Granada 2	DQ317572	Verdial Huévar 4	DQ117905		
Lechín de Granada 3	DQ640906	Verdial Huévar 5	DQ117906		
Lechín de Sevilla 1	DQ028766	Verdial Vélez-Málaga 1	DQ138358		
Lechín de Sevilla 2	DQ061976	Verdial Vélez-Málaga 2	DQ138359		
Lechín de Sevilla 3	DQ061977	Verdial Vélez-Málaga 3	DQ138360		
Lechín de Sevilla 4	DQ061978	Verdial Vélez-Málaga 4	DQ138361		
Loaime 1	DQ138339	Villalonga 1	DQ138351		
Loaime 2	DQ138340	Villalonga 2	DQ138352		
Loaime 3	DQ138341	Villalonga 3	DQ138353		
Loaime 4	DQ640903	Villalonga 4	DQ138354		
Lucio 1	DQ138362	Villalonga 5	DQ640907		

Accession numbers of the profilin cDNA sequences obtained after RT-PCR from pollen of five plant species: *Olea europaea*, *Betula pendula*, *Corylus avellana*, *Phleum pratense* and *Zea mays*.

**Figure 1:**
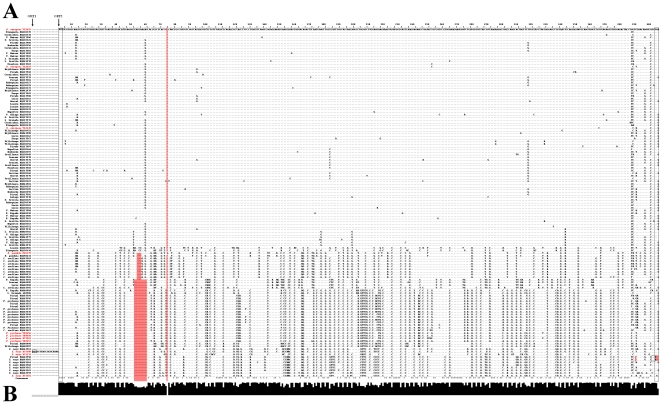
Multiple alignment of the nucleotide sequences of profilins. A) Two open reading frames (ORF) are indicated by vertical arrows. Numbering of the sequence positions begins at the ATG start codon from ORF2. Reference sequences of profilin from different species previously deposited in the GenBankTM/EMBL database were highlighted in red colour. Start and stop codons are indicated by colourless boxes. Significant deletions are depicted in red boxes. B) Sequence conservation index calculated with ClustalW software, 100% conservation corresponding to the maximal height of the bar at each position, and pointed out by an asterisk in the consensus.

**Figure 2:**
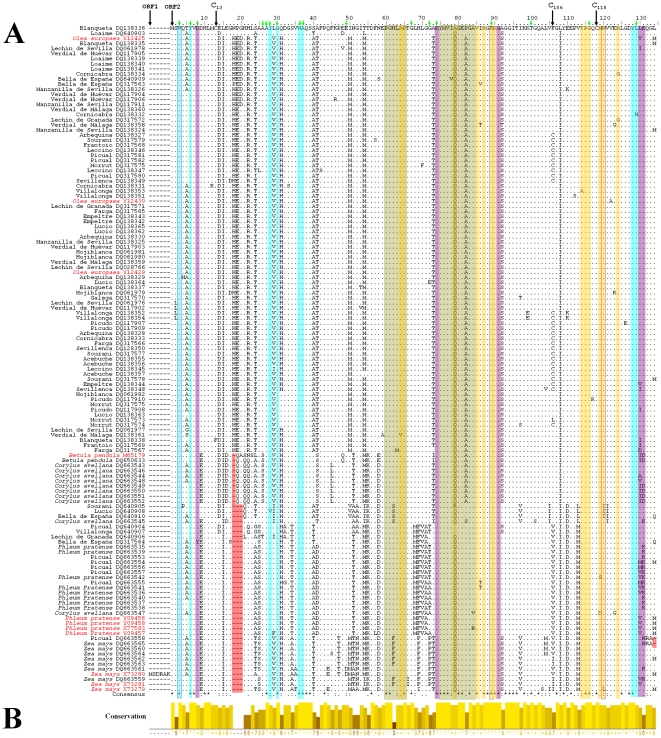
Multiple alignment of the deduced amino acid sequences of profilins. A) Key amino acid affecting protein folding and maintenance of the 3D structure, as well as Cys at positions 13, 106 and 118 are pointed by black and green arrows respectively at the top of the figure. Reference sequences of profilin from different species previously deposited in the GenBankTM/EMBL database were highlighted in red colour. Numbering of the sequence positions begins at the ATG start codon from ORF2. Significant deletions found in 1, 3 or 4 amino acids in the amino acid sequences are highlighted with red boxes. Amino acids integrating different ligand-binding regions are depicted with different coloured boxes: actin (orange), PLP (blue) and PIP (purple). Solvent filled plant-specific binding pockets are showed with green boxes. Alignment symbols: (.) same aa; (–) deletion. Consensus symbols: (*) same aa, (:) conservative change, (.) semi-conservative change, (•) non-conservative change. B) Sequence conservation index calculated by the Jalview program.

The full-length cDNA of the profilin sequences ranged between 393 bp (*Olea europaea*, DQ663558 or *Zea mays*, DQ663565) to 405 bp (*Olea europaea* Y12429, Y124230, Y12425). Peculiarities of a multiple sequence alignment of the profilins are shown in the Figure 1A, which also includes several profilins previously characterized and deposited in the GenBank databases. Variability along nucleotide sequences were calculated and depicted in Figure 1B. A remarkable feature was the presence of deletions in frame with protein translation in all the species analyzed, ranging from 3 nucleotides in *Betula verrucosa* (sequences DQ650633 and M65179) and *Corylus avellana* (DQ663543–4, DQ663546 and DQ663548–52), to 12 nucleotides in olive (Picual DQ663558) or maize (DQ663565). Deletions of 9 nucleotides were the most common feature in the remaining sequences with deletions. Protein sequences included polypeptides of 130, 131, 133 and 134 amino acids length (Figure 2A). The region along the profilin sequences with the lower conservation levels corresponded to the first 5′ half of the sequences, highly coinciding with the area of the main deletions (Figure 2B).

Frequent amino acid substitutions among the species were detected along the sequences. A total of 39 variable positions (variability index value, viv>3) were found in olive profilin (Table 2). The table 2 shows the variable positions and variability index in the olive profilin sequences, and in the rest of species. For all species (with the exception of birch), the most highly-variable residues were located in the N-terminal α-helix. The rest of variable positions were found all through the protein sequence. Different number of polymorphic positions was detected in Bet v 2, Cor a 2, Phl p 12 and Zea m 12 (5, 8, 3 and 4 positions respectively) (Table 2). Two key amino acids were affected by a high level of polymorphism in several species (positions 130 and 131 respectively, involved in the phosphoinositides lipids interaction), and the position 79, exclusively in olive tree (a position involved in actin interaction). Three key cysteines showed differential conservation as well, with the one in the position 106 displaying a high variability (viv>6).

**Table 2:** Summary of the polymorphic positions (viv>3) in the profilin sequences among the species analyzed in this work.

Sequence position	4	5	17	18	19	20	21	22	23	24	25	29	32	34	35	40	41	48	50	51	52
*Olea europaea*	3.06	5.47	3.10	4.62	3.55	4.16	3.13	3.59	-	5.71	3.38	3.55	3.13	3.35	-	3.23	3.42	3.06	3.55	3.46	3.20
*Betula pendula*	-	-	-	-	-	4.00	4.00	4.00	4.00	4.00	-	-	-	-	-	-	-	-	-	-	-
*Corylus avellana*	-	3.33	-	-	-	-	-	-	-	-	-	3.75	-	-	-	-	3.75	-	-	-	-
*Phleum pratense*	-	4.00	-	-	-	-	-	-	-	-	-	-	-	-	-	-	-	-	-	-	-
*Zea mays*	-	-	-	-	-	-	-	-	-	4.29	-	-	-	-	4.29	-	-	-	-	-	-

*Sequence position*	53	54	58	*62	70	72	73	*79**	*82	86	92	97	104	105	106	110	113	123	130***	131***	134
*Olea europaea*	3.06	4.26	3.50	3.13	3.23	5.51	-	3.13	3.91	3.10	3.42	4.26	3.10	3.10	6.64	3.60	3.46	3.06	5.45	3.20	4.36
*Betula pendula*	-	-	-	-	-	-	-	-	-	-	-	-	-	-	-	-	-	-	-	-	-
*Corylus avellana*	-	-	-	-	-	-	3.75	-	3.75	-	-	-	-	-	-	-	3.75	-	10.00	6.00	-
*Phleum pratense*	-	-	-	-	-	7.14	-	-	-	-	-	-	-	-	-	-	-	-	-	3.75	-
*Zea mays*	-	-	-	-	-	-	-	-	-	-	-	-	-	-	-	-	-	-	-	4.29	5.00

Polymorphic positions 62, 79 and 82 (asterisk), correspond to amino acids in the plant-specific binding pocket region. Position 79 (double-asterisk) is also one of the key amino acid for the profilin-actin interaction. Polymorphic positions 130 and 131 (triple-asterisk) correspond to important amino acids for the profilin-PIP interaction.

Concerning polymorphism at the family level, comparisons of the sequence identities between families showed that the variability between the *Oleaceae* and *Poaceae* families was 29.8 and 25.8% for nucleotide and amino acid sequences, respectively (they correspond to the most distant families), whereas *Oleaceae* and *Betulaceae* share higher identities, with variability percentages of 26.1 and 21.7% only (they are the most close-related families). The *Poaceae* and *Betulaceae* families showed 28.2 and 22.5% of variability, respectively (Table 3).

**Table 3:** Ranges of identity percentages calculated for both the nucleotide and the amino acid sequences of profilins in the species analysed.

	*Oleaceae Olea europaea*	*Corylus avellana*	*Betula pendula*	*Betulaceae* (*B. pendula*+*C. avellana*)	*Phleum pratense*	*Zea mays*	*Poaceae* (P. pratense+Z. mays)	
*Oleaceae Olea europaea*	71.8–100	72.3–98.4	73.6–81.9	72.3–98.4	74.4–97.7	70.4–92.9	70.4–100	Nucleotide
	75.4–100	76.3–96.1	74.4–86.5	74.4–96.1	76.1–100	74.2–100	74.2–100	Amino acid
	*Corylus avellana*	75.7–100	74.3–95.5	74.3–100	75.0–98.4	70.2–86.1	70.2–98.4	Nucleotide
		77.4–100	75.1–96.9	75.1–100	77.8–96.9	76.6–84.7	76.6–96.9	Amino acid
		*Betula pendula*	99.5–100	74.3–100	74.8–76.1	71.9–75.3	71.9–76.1	Nucleotide
			96.2–100	75.1–100	76.6–79.6	71.9–78.1	71.9–79.6	Amino acid
			*Betulaceae* (*B. pendula*+*C. avellana*)	74.3–100	74.1–98.4	70.2–86.1	70.2–98.4	Nucleotide
				75.1–100	76.6–96.9	74.4–84.7	74.4–96.9	Amino acid
				*P. pratense*	94.6–100	81.8–87.1	81.8–100	Nucleotide
					94.6–100	79.3–86.2	79.3–100	Amino acid
					*Z. mays*	79.9–100	79.3–100	Nucleotide
						84.6–100	80.1–100	Amino acid
						*Poaceae* (*P. pratense*+*Z. mays*	79.9–100	Nucleotide
							79.3–100	Amino acid

It also includes the data corresponding to the accumulated percentage for the comparisons between families. When comparing species, nucleotide sequence identity varied from 70.2% (*C. avellana*×*Z. mays*) to 100%, whereas identities in the amino acid sequences ranged from 71.9% (*B. pendula*×*Z. mays*) to 100%. The higher range of intra- specific variability was that of the *Olea europaea* L. nucleotide sequences (28.2%), whereas the profilin nucleotide sequences of *Betula pendula* showed the shorter range of variability (0.5%). Among the families, the percentage of nucleotide identity varied from 70.2% (*Betulaceae*×*Poaceae*) to 100% in several cases, while the percentages applied to the amino acid sequences ranged from 74.2% (*Oleaceae*×*Poaceae*) also to 100%. Intra- family variability in the nucleotide sequences ranged from 28.2% (*Oleaceae*) to 20.1% (*Poaceae*).

As regard to the polymorphism within the species analyzed, *Olea europaea* displayed the highest percentage of variability (28.2 and 24.6% for nucleotide and amino acid sequences respectively), while the lowest percentage of variability was found in *Phleum pratense* (20.1 and 20.7% for nucleotide and amino acid sequences, respectively). When polymorphism was analyzed between species, *Betula pendula* and *Phleum pratense* were the species with the lowest percentage of variability in nucleotide and amino acid sequences (1.3 and 3.0%, respectively). The most distant species in terms of sequence variability were *Olea europaea* and *Corylus avellana* with 21.6 and 19.8% of variability respectively (Table 3).

Additional pairwise alignments of more than 100 profilin sequences (data not shown) corresponding to a wide and representative number of plant species, showed a range of identity of 99.2–68.7% among plant reproductive profilin proteins, and 66.0–90.0% of identity among plant vegetative profilins. Furthermore, comparison of the plant reproductive profilins with profilins other than plant profilin proteins showed identity intervals: *P. polycephalum* (P22271) 45.8–36.0%, *D. discoideum* (P26199) 44.2–31.2%, *D. melanogaster* (P25843) 42.8–38.6%, *A. castellanii* (P68696), 42.7–34.3%, *S. cerevisiae* (P07274) 31.2–25.1%, *M. musculus* (P62962) 25.8–17.0%, *H. sapiens* (P07737) 25–17.7%; and virus (Q76ZN5) 12–6%.

A statistical analysis was performed to establish whether the differences in the variability at the level of both, nucleotide and amino acid sequence had statistical significance. The comparison of the variance for the distributions of variability showed significant differences for five species (F tests, p<<0.05). To analyze the variability differences between species, a *post hoc* range analysis between each pair of species was performed, assuming not normal distributions (Shapiro-Wilk tests, p<<0.05), and inequality of variances (Levene tests, p<<0.05). The table 4 shows the range analysis with the differences between pair of species determined by Games-Howell tests. At the level of nucleotide sequences, clear polymorphism differences were found between almost all pairs of species. Furthermore, amino acid sequences polymorphism revealed differences between many pairs of species.

**Table 4:** Statistical differences in polymorphism calculated among species for nucleotide and amino acid sequences.

Specie vs Specie	*Olea europaea*	*Betula pendula*	*Corylus avellana*	*Phleum pratense*	*Zea mays*	
*Olea europaea*		0.02p = 0.71	0.16^**^p = 0.00	0.12^**^p = 0.00	0.03p = 0.39	NUCLEOTIDE SEQUENCES
*Betula pendula*	0.83^**^p = 0.00		0.14^**^p = 0.00	0.11^**^p = 0.00	0.01p = 0.97	
*Corylus avellana*	0.15^**^p = 0.00	0.67^**^p = 0.00		0.32^**^p = 0.00	0.13^**^p = 0.00	
*Phleum pratense*	1.42^**^p = 0.00	0.58^**^p = 0.00	0.09p = 0.92		0.09^**^p = 0.00	
*Zea mays*	1.12^**^p = 0.00	0.28p = 0.42	0.38^**^p = 0.01	0.30p = 0.14		
	AMINO ACID SEQUENCES					

Comparisons in each box include the Game-Howell test and the level of significance (p<0.05). Statistical significative differences were stood out by double asterisk.

### Physicochemical properties and posttranslational modifications affected by the polymorphism

Most profilin sequences showed a calculated molecular weight and a calculated isoelectric point within the range of the profilins described in the literature and the NCBI database sequences (Table S1), with the exception of two protozoa basic profilins [30]. The average molecular weight was 14,334.40±144.01 Da. The average of the calculated isoelectric points (5.14±0.21) shows the acidic character of the profilin proteins (Table S1).

Most of the sequences exhibited hydrophilic character, as indicated by the negative average value (−0.15±0.06) of the calculated Grand index (GRAVY) [31]. Profilin protein sequences could be considered as stable proteins, at the light of the average aliphatic index of 80.03±3.62 [31], and the average stability index (27.95±2.07) [32], where values lower than 40 are considered as a stable protein (Table S1).

Post-translational motifs implicated in protein function regulation were analyzed by matching all the sequences with the PROSITE database [33]. Table S2 shows the variability in the potential N-myristoylation motifs in the olive, with a variable number of post-translational sites ranging between 1 to 4, including different motifs such as [^17^G(Q/L)hl(T/A)(A/S)^22^], [^30^GQdgSV^35^], [^33^GSvwAQ^38^], [^64^GMfvAG^69^], [^67^GLhlGG^72^], [^93^GGitSK^98^], where the numbers represent the position of the amino acids in the profilin sequence. Profilin sequences from birch showed 2 or 3 sites with different motifs such as [^17/19^GQqlAA^24^], [^32^GSvwAQ^37^] and [^66^GLhlGG^71^], whereas 2 sites were detected in timothy-grass sequences with two possible motifs, [^30^GTvwAQ^35^], and [^64^GMfvAT^69^]. In maize sequences, 1–2 myristoylation sites were detected, with the two possible motifs [^30^GA(t/a/v)wAQ^35^] and [^64^GLilGG^69^].

Larger homogeneity in the number of amidation motives was observed, with a unique predictive sequence [^84/87^rGKK^90^], present in all species. No glycosylation motifs were found in any of the profilin sequences, with the exception of the olive sequence DQ138337 (cv. Blanqueta) [^51^NGTM^54^].

A variable number of multi-optional phosphorilation sites were found, involving serine, threonine and tyrosine residues [34]. Serine residues susceptible of phosphorilation ranged between 0 to 2, threonine between 1 to 4 and tyrosine between 1 to 3 (Table S3). Finally, the analysis of variability in the phosphorylation motifs for Mitogen-activated protein (MAP) kinase was performed for all profilin sequences [35]. These motifs have been described in a large number of MAP kinase-interacting proteins, including profilin [36]. The table S2 also shows several changes for many profilin sequences, i.e. (**^79^**Q→A), (**^82^**A→P/S), (**^84^**A→V/R), (**^92^**S→A/T), (**^97^**I→V/S), which may affect the kinase interaction motif (KIM domain) [**^78^**IQGEAGAVIRGKKGSGGITIK**^98^**]. The major variability was found in the olive cultivar Bella de España, where all sequences were different. This kinase motif was also affected by the polymorphism at the level of species.

### Clustering analysis

**Figure 3:**
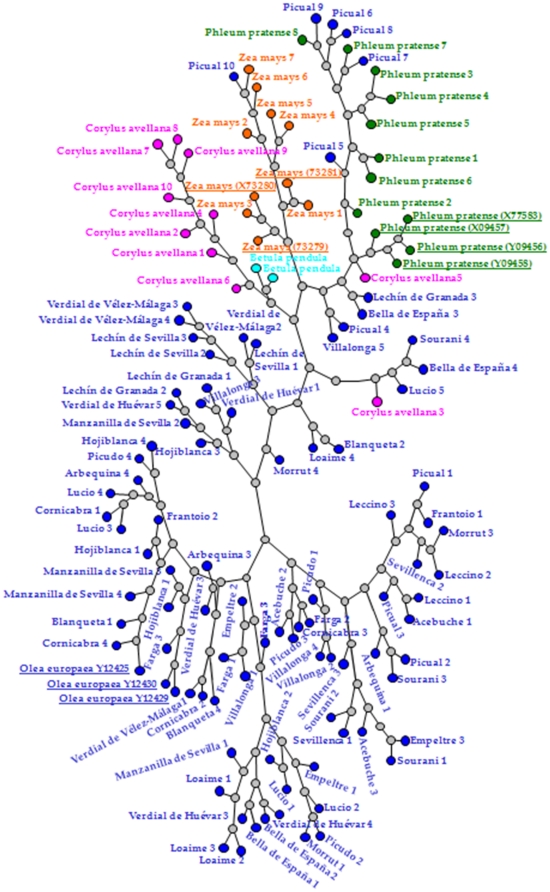
Phylogenetic analysis of profilin. Neighbor-Joining (NJ) method was used to perform a phylogenetic analysis of the deduced protein sequences of *O. europaea* L. (blue), *B. pendula* (magenta), *C. avellana* (pink), *P. pratense* (green), and *Z. mays* (orange) profilin. Reference sequences of profilin from different species previously deposited in the GenBankTM/EMBL database were highlighted by underline.

Phylogenetic analysis was performed in order to determine the relationships between profilin sequences in different species, and to infer the evolutive trends among the wide representation of the olive germplasm (Figure 3). When all the sequences were analyzed together, a clear separation between the five species was detected. Several exceptions for olive profilin, and two sequences for hazel were found, with sequences of these species located in a tree branch belonging to timothy-grass and maize (Figure 3).

Furthermore, the analysis of the individual species showed no clear relationships for olive cultivars, where the sequences were mixed and distributed around branches of the tree.

## Discussion

### Multiple profilin isovariants as a result of the sequence polymorphism reveal their multifunctionality

The presence of protein variants is one of the most widespread properties in higher plants, mainly as the result of the occurrence of multigene families [6]. Moreover, different mechanisms rising protein variability have been described such as single nucleotide polymorphisms (SNP) and post-translational modifications. The existence of profilin isovariants in pollen has been described in *Zea mays* [23], *Triticum aestivum* [24], *Nicotiana tabacum* [25], soybean [37] and *Olea europaea* [26]. In addition, microheterogeneities have been identified in different sources, including foods [38], grasses and tree pollen [26], [27]. Alternative splicing is also another mechanism to generate protein variability, i.e. profilin II in mouse [39], apple [38] and *Arabidopsis*, where sequence introns are implicated in the constitutive expression of profilin in most vegetative tissues, exhibiting functionally differential properties in reproductive tissues [40].

We have performed an extensive analysis of profilin sequence polymorphism, which includes the first report of such variability in hazel pollen profilin, as well as the largest number of olive pollen cultivars ever analyzed. Similarly to what occurs in other plant species, profilin variability in the 5 species analyzed might be risen by multiple gene copies [6]. In addition, sequence polymorphism described here would come up as new mechanism to generate variability, more concretely in plant species such as olive, with a wide germplasm (more than 2000 cultivars around the world) [41], suffering different stress or physiological conditions. Thus, sequence polymorphism is affecting protein functional and regulatory motifs, as reported here for Ser/Thr or Tyr phosphorylation, might be able to regulate profilin activity and those processes implicated by differentiated dynamics.

The complex expression patterns and functional diversity of profilin family members in other plant species has been correlated with the existence of specific cellular functions [12] and its complex regulation [21]. Our results have shown a wide variability generated through olive germplasm that would be enough to afford the functional requirements, in terms of actin dynamics and signal transduction, buffering different stress and physiological conditions [29], and to provide an additional mechanism to protect cells from loss-of-functions with multiple forms of profilins, with overlapping and specific functions. Protein isoforms with unique functions could be required at different times or locations, while overlapping expression of different cytoskeletal proteins and functionally distinctive isoforms are required at the same time in the same tissue or cell [42]. Our result confirms that profilin isoforms exhibit different Mw and pI as result of the polymorphism. Birch and timothy grass pollen profilin have shown a rather acidic pI [43]. In addition, *Acanthamoeba* [44], *Dictyostelium* [45] and humans [46] have several profilin isoforms with differences in sequence, pI, function and expression pattern. Maize pollen profilins also have different tissue expression [23] and function [42].

Deletions seem to be an additional or alternative mechanism to generate profilin isovariants with differential physicochemical properties, e.g. the human PROF III and a virus profilin homologue [47]. We have shown in this work the presence of deletions in 47 sequences of profilin, which also exhibit differential properties (MW, pI, post-translational modification sites), supporting the existence of this mechanism to generate profilin variability.

Nevertheless, it is worthy to mention that many of the profilin sequences described here exhibited multi-optional post-translational sites generated by polymorphism, e.g. new or missed residues of serine or threonine [48], [34], or tyrosine implicated in phosphorylation and protein regulation by potential differential interactions with different partners such as PLP or PLP-proteins [49], [50].

Other evidence supporting the existing polymorphism as a mechanism to generate multiple forms of profilins in plants come up with its co-evolution with different forms of actin and other ABPs, more precisely profilin-interacting proteins. There are evidences of different actin isoforms as the result of multiple genes in *Arabidopsis* [51] and soybean [52], and strong evidences of functional differences among actin classes, e.g. isoelectric points of the plant actins vary over a relatively wide [53], [54].

Plant genomes encode large vegetative and reproductive classes of actin and ABP gene families [51], with different ABP isovariants, e.g. profilin, with class-specific preferential interaction between the actin and profilin isovariants in plants [55]. Plant actin proteins have an unusually large number of non-conservative amino acid substitutions (6 to 10%) mapping to the surface of the molecule [51], which have a significant effect on protein–protein interactions; i.e. profilin and thymosin [56]. Fourteen additional families of ABPs encoded by multiple genes are implicated in actin dynamics, where formin is the most prominent actin nucleator and profilin interacting protein family. This profilin-interacting protein is responsible for the assembly and elongation of unbranched actin filaments. Formin family is integrated at least by 21 genes in *Arabidopsis* [57], and shows a sequence identity range between 15 to 55% in plants [58]. This high variability would indirectly regulate the number of isovariants of interacting partners like profilin.

Finally, profilin variability is also reflected in their multiple subcellular localization, functions and regulation under different conditions. It is generally accepted that profilins have a cytoplasmatic localization [59]. They are preferentially associated to plasma membrane domains, and differentially located within developing microspores [60]. Other possible locations are amiloplasts [61], and generative and vegetative nuclei in pollen [62], since different profilin partners of nuclear localization have been found like PIP_2_ [63] and actin [64]. Chloroplast is one of the last and newly characterized profilin locations, as a result of the interaction with the Chloroplast Unusual Positioning 1 (CHUP1) protein [65].

### Profilin polymorphism as the result of post-translational modifications and changes in partners-binding regions is also an important factor affecting multifunctionality

Profilins have surface-exposed binding areas for actins [66], poly-L-proline (PLP) proteins [67], and phosphoinositide lipids [68], [69]. The interacting motives have been identified in many proteins, including plant profilins [70]. The affinity for the different ligands differs by orders of magnitude between species and even isoforms [71], [72]. Extreme examples include a minor splicing form in mouse that has been reported not to bind to G-actin [73], and Vaccinia virus profilin, which does not bind to PLP [74]. Differential properties as a result of sequence polymorphism in profilin isovariants would bring up a range of interacting affinities for ATP, actin and PLP [23], [75]. In this regard, sequence variability would constitute a mechanism able to increase the possibilities for plant responses to multiple stress and physiological conditions, which would be mediated by the transduction of external signals through actin dynamics [76].

The actin molecule exhibits a wide surface involved in profilin interaction (2,250 Å^2^) [66], and a large number of non-conservative amino acid substitutions have been exhibited in various plant actin molecules [51]. In addition, our results confirm that the N-terminal region of profilin involved in the interaction with actin is non-conservative, with a large number of variable residues located close to the actin-interacting area in the profilin surface. This variability in the actin-binding surface has been also reported in different species [70].

Furthermore, the so-called “plant specific binding pocket” of the profilins analyzed here, which is also implicated in the actin interaction, contains two highly variable residues (H62 and Q79). Our results indicate that the polymorphism surrounding the actin-interacting area, as well as the variability of the residues directly implicated in this interaction, would affect the binding properties of profilin isovariants. In addition, the variability affecting residues involved in phosphorylation within the actin interaction area might also play a regulatory role in the properties of this interaction with different profilins [77].

PLP-binding stretches play a major role for profilin interaction with proline-rich proteins [78]. The affinity of profilin interactions is quite variable [71], [72], and it is also regulated through phosphorylation [77], [50]. Profilin sequence polymorphism affecting phosphorylation sites, precisely tyrosines 6 and 128 in olive cultivars, would be a major mechanism to regulate the affinity of profilin-PLPs interactions, especially under different cellular processes and environmental stresses [49], [50]. In addition, a new domain located around tyrosine 75 has been also implicated in profilin-PLP and PI3K interactions. This interacting area, integrated by a characteristic plant pocket is defined by three regularly spaced aromatic residues highly conserved among profilin sequences, and followed by a pattern of three residues susceptible of phosphorylation (such as positions T66, Y75 and Y109). The analysis of polymorphism of this region in olive profilins has shown a variable number of combinations of phosphorylation sites among these three positions, which could be phosphorylated *in vivo* [49], probably because they are exposed in the protein surface and accessible to the solvent [70]. Phosphorylations within PLP domains might be a fundamental regulatory process, able to generate isovariants with differential interacting properties [42]. It was previously demonstrated that isoforms of profilins are generated by tyrosine phosphorilation in different tissues of *Phaseolus vulgaris*. Furthermore, these modifications would mimic physicochemical properties such as different maize profilin isovariants do it, raising the possibility that individual phosphorylated isoforms might display specific roles in different tissues, since tyrosine phosphorylation in poly-L-proline-binding regions inhibits binding to phosphoinositide 3-kinase in *Phaseolus vulgaris* [50].

The profilin region interacting with lipid phosphoinositides is divided in two areas of the protein surface. Changes in residues belonging to the actin-interacting area can either increase or decrease the affinity of profilin to PIP [79]. A second binding site for PIP_2_ is overlapping with the poly-L-proline-binding site at the C-terminal region of the profilin molecule [39], [80]. This area allows the molecular interaction and a possible competition between PIP_2_ and the PLP ligands at the C-terminal site. The profilin sequences analyzed here have shown a high variability in this area, concretely in the positions Leu130 and Glu131, which are directly implicated in the PIP interaction. This variability would make it possible to regulate the interaction affinity of PIP lipid in profilin isovariants [69], [71], [80], as well as the interaction with other lipid phosphoinositides (PI_(3,4)_P_2_ and PI_(3,4,5)_P_2_) [81]. These differential affinities to several PIP molecules would be a mechanism to regulate differential signal transduction, buffering different stress and physiological signals throughout actin rearrangements. Furthermore, changes in the PIP-PLP binding overlapping region would make a second competitive mechanism to control interactions with different poly-L-proline rich proteins.

Both overlapping regions together, actin-PIP-PLP might be a regulatory mechanism for a positive or negative interaction with different ligands, under different cellular environments, e.g. phosphorylation in serine 92 by protein kinase C zeta has been reported to increase the affinity for G-actin and PLP, while the interaction with PIP_2_ remained unaltered [77]. This serine residue in highly variable in olive cultivars and timothy-grass sequences, which constitute another example to support that sequence polymorphism is a mechanism generating profilin variability, regulating the differential properties of interaction with actin and PLP [71], [82].

### Conclusion

The study presented here has revealed the possible functional and regulatory consequences of sequence polymorphism in pollen profilins. This polymorphism might represent a mechanism to generate multiple profilin isovariants among species, the germplasm of a particular species, their tissue or even their subcellular localization. These isovariants, exhibiting a wide range of physicochemical differences as well as differences in profilin-ligand binding properties, could have a direct influence in the cellular dynamics and the regulatory processes orchestrated by actin cytoskeleton, leading to more robust and a wide range of responses of cells to different physiological and stress conditions. In addition, the co-existence of different profilin variants in the same cell would allow more complex processes of signals integration through proteins (ABPs) interaction networks and cytoskeletal rearrangements.

Further research will unravel the effects of the polymorphism in different structures (folding) of the profilin isoforms, and its influence in the interaction with different ligands and in the cellular dynamics. Overall, the knowledge gained will help provide a comprehensive understanding on the stunning variety of functions of these small, ubiquitous proteins.

## Materials and Methods

Olive (*Olea europaea* L.) pollen was individually collected during May and June from olive trees of 24 different cultivars, grown in different olive germplasm collections in Spain (CIFA “Alameda del Obispo”, Córdoba, CIFA “Venta del Llano”, Jaén, Olive Culture Museum, Baeza, Jaén, and Estación Experimental del Zaidín, CSIC, Granada). Pollen samples were collected in large paper bags by vigorously shaking the inflorescences, sequentially sieved through 150 and 50 µm mesh filters to eliminate debris and maintained at −80°C. Pollen from *Betula verrucosa* var. Laciniata and *Corylus avellana* var. Avellana was collected from well-identified trees at the Botanical Garden of the University of Bonn (Germany) using the same procedure described above. Commercially available pollen (Allergome, Sweden) was used in the case of *Phleum pratense* var. Pratense and *Zea mays* var. Birko.

### RT-PCR, Cloning and sequencing of profilin transcripts

Total RNA was isolated from 100 mg pollen samples of each cultivar/species by using the RNeasy Plant Total RNA kit (Qiagen). cDNA synthesis was carried out by using Superscript II reverse transcriptase (Invitrogen) and a poly-dT adaptor as a primer, following manufacturer's indications. PCR amplifications were carried out from 50–100 ng of the template cDNA, by using 0.2–0.5 µM of each one of the following degenerated primers [5′-AGAGAATTCCATATGTCGTGGCA(A/G)(A/G)CGTACGT-3′] (forward) and [5′-AGAAAGCTT(C/T)TACA(G/T)GCC(C/T)TGTTCA(C/G/T)(A/C/G)AGGTA-3′] (reverse), 1 µl (2.5 U) of the PfuUltra High-Fidelity DNA Polymerase (Stratagene), 250 µM each dNTP, final reaction buffer at 1X, and ultrapure water up to 50 µl of final reaction volume. PCR mixtures were subjected to the following conditions in a Biometra T-Gradient Thermocycler (Biotron, Germany): initial heating step at 95°C for 5 minutes, denaturation at 94°C for 30 sec, annealing at 56°C for 45 sec, and extension at 72°C for 1 min. A final extension step of 10 min at 72°C was included after 30 cycles. After analyzing the PCR products by agarose gel electrophoresis, bands (405 bp) were excised and purified from gel with the Gel Purification Kit (Qiagen). Purified fragments were ligated into the pGEM-T easy Vector (Promega) and used to transform *Escherichia coli* DH_5α_ competent cells (Stratagene) according to the manufacturer's instructions. Variable number (1–10) clones were sequenced.

### Polymorphism analysis of profilin sequences

Both nucleotide and deduced amino acid sequences obtained in the current work were searched for identity by the nucleotide-nucleotide BLAST (blastn) and amino acid BLAST (Blastp) programs [83], respectively. A multiple sequence alignment and subsequent analysis was performed using ClustalW software [84], based on Blosum62 matrix (BLOck SUbstitution Matrix) [85], and viewed using the Jalview viewer 2.2 [86]. The Bioedit v 7.0.5.3 [87] software was used to calculate the sequence identity matrices.

In order to assess the variability present in nucleotide sequences, the alignment was used to calculate an entropy plot for each specie [88], [89] by measuring of the lack of “bits of information content” at each position in the alignment. For amino acid sequences, variability was calculated as the number of different residues occurring at each position of the alignment divided by the frequency of the most common one [90]. The numerical obtained regarding nucleotide and amino acid variability were calculated and summarized as intervals of identity percentages within families, species and cultivars throughout identity matrices.

### Physicochemical properties and post-translational modification motifs

Physicochemical properties of the profilin sequences were analyzed by using the ExPASy Proteomics Server. The ProtParam tool [91] was implemented to calculate the MW/pI of the different profilins, as well as instability index, aliphatic index and grand average of hydropathicity (GRAVY).

Profilin consensus patterns were checked for each original sequence and further analysis were performed to highlight the presence of functional motifs by using the PROSITE database [92]. Biologically meaningful motifs, susceptible of posttranslational modifications were derived from multiple alignments and the ScanProsite program [93], from the Expert Protein Analysis System (ExPASy) proteomics server of the Swiss Institute of Bioinformatics [94], as well as phosphorilation motives were analyzed by using NETPhos v1.0 [95] and NETPhosK v1.0 [96].

### Phylogenetic analysis of profilin sequences

Profilin proteins from 5 plant species were used to generate phylogenetic trees using ClustalW [84]. The alignment was created using the Blosum62 matrix (BLOck SUbstitution Matrix) [85], multiple alignment gap opening/extension penalties of 10/0.5 and pairwise gap opening/extension penalties of 10/0.1. These alignments were adjusted using Bioedit V7.0.5.3 [86]. Portions of sequences that could not be reliably aligned were eliminated. Phylogenetic tree was generated by the neighbor-joining method (NJ) [97], and the branches were tested with 1,000 bootstrap replicates. The three was visualized using Treedyn program [98].

### Statistical analysis of polymorphism

Statistical analysis was performed by using the SPSS v.18 statistical software package. A General comparison among multiple sample groups was performed throughout one-way analysis of variance (*One-way* ANOVA) on the basis of the Fisher-Snedecor distribution test (α = 0.05 significance value) [99]. Normality and variances homogeneity of the data collection were checked by the Shapiro-Wilk test (α = 0.05 significance value) [100] and the Levene test (α = 0.05 significance value) [101], respectively, and *post hoc* range probes and pair of species comparisons were carried out with the parametric test of Games-Howell (α = 0.05 significance value) [102].

## Supporting Information

Table S1 **Physic-chemical properties deduced from the profilin sequences.** Different physic-chemical parametes were calculated for the amino acid sequences of profilin from the five species studied. Parameters were: molecular weight (PM), isoelectric point (pI), extinction molar coefficient (C.E.M.) at 280 nm (M^−1^ cm^−1^) (-SH-SH-/-S-S-), instability index, GRAVY, and aliphatic index.
(DOC)

Table S2 **Analysis of the polymorphism affecting posttranslational modification motifs in the profilin sequences.** The most representative and important posttranslational modifications were examined for the profilin amino acid sequences. These motifs included: N-myristoylation, amidation, phosphorylation by MAP kinase, N-glycosilation and targeting signal for microsomal bodies.
(DOC)

Table S3 **The polymorphism of putative phosphorylation residues.** Serine, threonine and tyrosine residues of the profilin sequences were analyzed. Multiple combinations of residues for susceptible phosphorylation were found among profilin sequences.
(DOC)
