# Supplementary material for: Characterization of Profilin Polymorphism in Pollen with a Focus on Multifunctionality
Source: PLoS One. 2012 Feb 14;7(2):e30878. doi: 10.1371/journal.pone.0030878 (PMC3279341; doi:10.1371/journal.pone.0030878)
Supplement: Table S1 — Physic-chemical properties deduced from the profilin sequences. Different physic-chemical parametes were calculated for the amino acid sequences of profilin from the five species studied. Parameters were: molecular weight (PM), isoelectric point (pI), extinction molar coefficient (C.E.M.) at 280 nm (M−1 cm−1) (-SH-SH-/-S-S-), instability index, GRAVY, and aliphatic index. (DOC) [file pone.0030878.s001.doc]

**Table S1. Physic-chemical properties deduced from the profilin sequences.**

| **GeneBank Accession N°** | ***Olea europaea* L.**  **Cultivar** | **MW**  **(Da)** | **Ip** | **N° Amino acids** | **C.E.M.**  **(M^-1^cm^-1^)** | **Instability Index** | **GRAVY** | **Aliphatic Index** |  | **GeneBank Accession N°** | ***Olea europaea* L.**  **Cultivar** | **MW**  **(Da)** | **Ip** | **N° Amino acids** | **C.E.M.**  **(M^-1^cm^-1^)** | **Instability Index** | **GRAVY** | **Aliphatic Index** |
| --- | --- | --- | --- | --- | --- | --- | --- | --- | --- | --- | --- | --- | --- | --- | --- | --- | --- | --- |
| **Y12425** | **-** | **14489.44** | **5.06** | **134** | **17085/16960** | **27.96** | **-0.207** | **75.67** |  | **DQ663554** | **Picual** | **14195.37** | **5.22** | **131** | **17085/16960** | **27.90** | **-0.097** | **76.72** |
| **Y12429** | **-** | **14427.39** | **5.21** | **134** | **17085/16960** | **26.52** | **-0.172** | **79.33** |  | **DQ663555** |  | **14077.22** | **5.43** | **131** | **17085/16960** | **27.40** | **-0.079** | **77.48** |
| **Y12430** | **-** | **14399.34** | **5.21** | **134** | **17085/16960** | **26.52** | **-0.190** | **77.91** |  | **DQ663556** |  | **14177.34** | **5.22** | **131** | **17085/16960** | **27.90** | **-0.082** | **79.69** |
| **DQ138355** | **Acebuche** | **14383.36** | **5.21** | **134** | **17085/16960** | **26.52** | **-0.175** | **79.33** |  | **DQ663557** |  | **14145.28** | **5.22** | **131** | **17085/16960** | **27.68** | **-0.065** | **81.91** |
| **DQ138356** |  | **14383.36** | **5.21** | **134** | **17085/16960** | **26.52** | **-0.175** | **79.33** |  | **DQ663558** |  | **14163.27** | **5.24** | **130** | **17085/16960** | **28.30** | **-0.120** | **75.00** |
| **DQ138357** |  | **14353.27** | **5.21** | **134** | **17085/16960** | **26.73** | **-0.194** | **79.33** |  | **DQ117907** | **Picudo** | **14455.42** | **5.07** | **134** | **17085/16960** | **27.96** | **-0.198** | **79.33** |
| **DQ138327** | **Arbequina** | **14413.38** | **5.21** | **134** | **17085/16960** | **26.52** | **-0.193** | **78.52** |  | **DQ117908** |  | **14418.38** | **5.06** | **134** | **17085/16960** | **31.23** | **-0.169** | **79.33** |
| **DQ138328** |  | **14383.36** | **5.21** | **134** | **17085/16960** | **26.52** | **-0.175** | **79.33** |  | **DQ117909** |  | **14383.36** | **5.21** | **134** | **17085/16960** | **26.52** | **-0.175** | **79.33** |
| **DQ138329** |  | **14436.40** | **5.36** | **134** | **17085/16960** | **28.29** | **-0.170** | **79.33** |  | **DQ117910** |  | **14418.43** | **5.24** | **134** | **17085/16960** | **27.88** | **-0.178** | **79.33** |
| **DQ138330** |  | **14427.39** | **5.21** | **134** | **17085/16960** | **26.52** | **-0.172** | **79.33** |  | **DQ138348** | **Sevillenca** | **14369.33** | **5.21** | **134** | **17085/16960** | **29.80** | **-0.166** | **78.58** |
| **DQ317563** | **Bella de España** | **14431.38** | **4.92** | **134** | **17085/16960** | **29.25** | **-0.186** | **78.58** |  | **DQ138349** |  | **14471.42** | **5.06** | **134** | **17085/16960** | **27.96** | **-0.216** | **78.58** |
| **DQ317564** |  | **14444.38** | **5.06** | **134** | **17085/16960** | **29.39** | **-0.178** | **78.58** |  | **DQ138350** |  | **14383.36** | **5.21** | **134** | **17085/16960** | **26.52** | **-0.175** | **79.33** |
| **DQ640909** |  | **14211.30** | **5.23** | **131** | **18575/18450** | **31.07** | **-0.103** | **78.93** |  | **DQ317577** | **Sourani** | **14383.36** | **5.21** | **134** | **17085/16960** | **26.52** | **-0.175** | **79.33** |
| **DQ640910** |  | **14009.96** | **4.87** | **131** | **17085/16960** | **33.53** | **-0.066** | **90.15** |  | **DQ317578** |  | **14401.39** | **5.21** | **134** | **17085/16960** | **26.52** | **-0.189** | **76.42** |
| **DQ138335** | **Blanqueta** | **14503.46** | **5.06** | **134** | **17085/16960** | **27.96** | **-0.210** | **76.42** |  | **DQ317579** |  | **14353.28** | **5.21** | **134** | **17085/16960** | **28.52** | **-0.220** | **78.58** |
| **DQ138336** |  | **14249.17** | **5.04** | **134** | **17085/16960** | **28.70** | **-0.134** | **83.73** |  | **DQ640905** |  | **14022.06** | **5.04** | **131** | **17085/16960** | **32.88** | **-0.023** | **92.37** |
| **DQ138337** |  | **14415.34** | **5.21** | **134** | **17085/16960** | **26.52** | **-0.211** | **76.42** |  | **DQ117902** | **Verdial Huevar** | **14439.45** | **5.21** | **134** | **17085/16960** | **25.64** | **-0.140** | **81.49** |
| **DQ138338** |  | **14501.46** | **5.21** | **134** | **16960/16960** | **29.28** | **-0.184** | **78.58** |  | **DQ117903** |  | **14427.39** | **5.21** | **134** | **17085/16960** | **26.52** | **-0.172** | **79.33** |
| **DQ138331** | **Cornicabra** | **14454.40** | **5.40** | **134** | **17085/16960** | **28.76** | **-0.219** | **79.33** |  | **DQ117904** |  | **14515.46** | **5.06** | **134** | **17085/16960** | **27.96** | **-0.214** | **78.58** |
| **DQ138332** |  | **14481.40** | **5.36** | **134** | **17085/16960** | **27.44** | **-0.243** | **75.67** |  | **DQ117905** |  | **14485.43** | **5.06** | **134** | **17085/16960** | **27.96** | **-0.196** | **78.58** |
| **DQ138333** |  | **14383.36** | **5.21** | **134** | **17085/16960** | **26.52** | **-0.175** | **79.33** |  | **DQ117906** |  | **14543.47** | **5.06** | **134** | **17085/16960** | **31.08** | **-0.219** | **78.58** |
| **DQ138334** |  | **14386.30** | **4.91** | **134** | **17085/16960** | **27.96** | **-0.165** | **79.33** |  | **DQ138358** | **Verdial Málaga** | **14387.33** | **5.38** | **134** | **17085/16960** | **23.98** | **-0.206** | **75.67** |
| **DQ138342** | **Empeltre** | **14427.39** | **5.21** | **134** | **17085/16960** | **26.52** | **-0.172** | **79.33** |  | **DQ138359** |  | **14427.39** | **5.21** | **134** | **17085/16960** | **26.52** | **-0.172** | **79.33** |
| **DQ138343** |  | **14427.39** | **5.21** | **134** | **17085/16960** | **26.52** | **-0.172** | **79.33** |  | **DQ138360** |  | **14457.42** | **5.21** | **134** | **17085/16960** | **26.52** | **-0.191** | **78.58** |
| **DQ138344** |  | **14369.33** | **5.21** | **134** | **17085/16960** | **26.52** | **-0.172** | **78.58** |  | **DQ138361** |  | **14413.41** | **5.38** | **134** | **17085/16960** | **29.80** | **-0.134** | **80.75** |
| **DQ317565** | **Farga** | **14427.39** | **5.21** | **134** | **17085/16960** | **26.52** | **-0.172** | **79.33** |  | **DQ138351** | **Villalonga** | **14417.36** | **5.21** | **134** | **17085/16960** | **25.08** | **-0.166** | **79.33** |
| **DQ317566** |  | **14383.36** | **5.21** | **134** | **17085/16960** | **26.52** | **-0.175** | **79.33** |  | **DQ138352** |  | **14409.44** | **5.21** | **134** | **17085/16960** | **23.14** | **-0.140** | **82.24** |
| **DQ317567** |  | **14417.33** | **5.06** | **134** | **17085/16960** | **27.44** | **-0.222** | **74.25** |  | **DQ138353** |  | **14397.37** | **5.21** | **134** | **17085/16960** | **28.58** | **-0.154** | **80.07** |
| **DQ317568** | **Frantoio** | **14413.38** | **5.21** | **134** | **17085/16960** | **26.52** | **-0.193** | **78.58** |  | **DQ138354** |  | **14409.44** | **5.21** | **134** | **17085/16960** | **23.14** | **-0.140** | **82.24** |
| **DQ317569** |  | **14427.39** | **5.21** | **134** | **17085/16960** | **29.80** | **-0.167** | **79.33** |  | **DQ640907** |  | **14038.17** | **5.04** | **131** | **17085/16960** | **30.73** | **0.015** | **83.44** |
| **DQ317570** | **Galega** | **14415.34** | **5.21** | **134** | **17085/16960** | **27.15** | **-0.211** | **76.42** |  |  |  |  |  |  |  |  |  |  |
| **DQ061979** | **Hojiblanca** | **14484.49** | **5.39** | **134** | **17085/16960** | **30.17** | **-0.199** | **79.33** |  | **GeneBank Accession N°** | **Specie** | **MW**  **(Da)** | **Ip** | **N° Amino acids** | **C.E.M.**  **(M^-1^cm^-1^)** | **Instability Index** | **GRAVY** | **Aliphatic Index** |
| **DQ061980** |  | **14427.39** | **5.21** | **134** | **17085/16960** | **26.52** | **-0.172** | **79.33** |  |  |  |  |  |  |  |  |  |  |
| **DQ061981** |  | **14427.39** | **5.21** | **134** | **17085/16960** | **26.52** | **-0.172** | **79.33** |  | **M65179** | ***Betula pendula*** | **14253.21** | **5.02** | **133** | **17085/16960** | **26.40** | **-0.106** | **84.29** |
| **DQ061982** |  | **14418.38** | **5.06** | **134** | **17085/16960** | **27.96** | **-0.175** | **79.33** |  | **DQ650633** |  | **14278.26** | **5.02** | **133** | **-** | **27.85** | **-0.123** | **84.29** |
| **DQ138345** | **Leccino** | **14397.38** | **5.21** | **134** | **17085/16960** | **27.79** | **-0.172** | **80.07** |  | **DQ663543** | ***Corylus avellana*** | **14203.15** | **4.89** | **133** | **17085/16960** | **30.33** | **-0.141** | **85.04** |
| **DQ138346** |  | **14413.38** | **5.21** | **134** | **17085/16960** | **26.52** | **-0.193** | **78.58** |  | **DQ663544** |  | **14161.07** | **4.89** | **133** | **17085/16960** | **30.33** | **-0.156** | **82.86** |
| **DQ138347** |  | **14395.36** | **5.21** | **134** | **17085/16960** | **27.44** | **-0.205** | **80.75** |  | **DQ663545** |  | **14083.16** | **5.41** | **131** | **17085/16960** | **30.35** | **-0.007** | **93.13** |
| **DQ317571** | **Lechín de Granada** | **14427.39** | **5.21** | **134** | **17085/16960** | **26.52** | **-0.172** | **79.33** |  | **DQ663546** |  | **14203.15** | **4.89** | **133** | **17085/16960** | **30.33** | **-0.141** | **85.04** |
| **DQ317572** |  | **14358.28** | **5.04** | **134** | **17085/16960** | **26.52** | **-0.160** | **78.58** |  | **DQ663547** |  | **14087.20** | **4.90** | **131** | **17085/16960** | **29.03** | **-0.004** | **84.89** |
| **DQ640906** |  | **14094.27** | **5.06** | **131** | **17085/16960** | **26.67** | **0.020** | **84.89** |  | **DQ663548** |  | **14189.12** | **4.89** | **133** | **17085/16960** | **30.33** | **-0.138** | **84.29** |
| **DQ028766** | **Lechín de Sevilla** | **14427.39** | **5.21** | **134** | **17085/16960** | **26.52** | **-0.172** | **79.33** |  | **DQ663549** |  | **14219.15** | **4.87** | **133** | **17085/16960** | **28.88** | **-0.155** | **84.29** |
| **DQ061976** |  | **14453.48** | **5.21** | **134** | **17085/16960** | **25.01** | **-0.138** | **82.24** |  | **DQ663550** |  | **14219.15** | **4.87** | **133** | **17085/16960** | **28.88** | **-0.155** | **84.29** |
| **DQ061977** |  | **14413.37** | **5.21** | **134** | **17085/16960** | **25.88** | **-0.189** | **78.58** |  | **DQ663551** |  | **14219.15** | **4.87** | **133** | **17085/16960** | **28.88** | **-0.155** | **84.29** |
| **DQ061978** |  | **14455.45** | **5.20** | **134** | **17085/16960** | **28.94** | **-0.133** | **81.49** |  | **DQ663552** |  | **14219.15** | **4.87** | **133** | **17085/16960** | **28.88** | **-0.155** | **84.29** |
| **DQ138339** | **Loaime** | **14485.43** | **5.06** | **134** | **17085/16960** | **27.96** | **-0.196** | **79.33** |  | **X77583** | ***Phleum pratense*** | **14235.34** | **5.07** | **131** | **17085/16960** | **27.56** | **-0.141** | **77.40** |
| **DQ138340** |  | **14485.43** | **5.06** | **134** | **17085/16960** | **27.96** | **-0.196** | **79.33** |  | **Y09456** |  | **14150.23** | **4.92** | **131** | **17085/16960** | **26.91** | **-0.093** | **78.17** |
| **DQ138341** |  | **14485.43** | **5.06** | **134** | **17085/16960** | **27.96** | **-0.196** | **79.33** |  | **Y09457** |  | **14164.26** | **4.92** | **131** | **17085/16960** | **25.73** | **-0.076** | **78.93** |
| **DQ640903** |  | **14274.22** | **5.00** | **134** | **17085/16960** | **25.85** | **-0.116** | **86.64** |  | **Y09458** |  | **14150.23** | **4.92** | **131** | **17085/16960** | **26.91** | **-0.093** | **78.17** |
| **DQ138362** | **Lucio** | **14427.39** | **5.21** | **134** | **17085/16960** | **26.52** | **-0.172** | **79.33** |  | **DQ663535** |  | **14159.31** | **5.22** | **131** | **17085/16960** | **27.25** | **-0.068** | **82.67** |
| **DQ138363** |  | **14448.41** | **5.06** | **134** | **17085/16960** | **27.96** | **-0.193** | **78.58** |  | **DQ663536** |  | **14115.25** | **5.22** | **131** | **17085/16960** | **27.68** | **-0.046** | **62.67** |
| **DQ138365** |  | **14427.39** | **5.21** | **134** | **17085/16960** | **26.52** | **-0.172** | **79.33** |  | **DQ663537** |  | **14159.31** | **5.22** | **131** | **17085/16960** | **27.25** | **-0.068** | **82.67** |
| **DQ138364** |  | **14499.46** | **5.07** | **134** | **17085/16960** | **25.67** | **-0.196** | **79.33** |  | **DQ663538** |  | **14189.33** | **5.22** | **131** | **17085/16960** | **27.25** | **-0.087** | **81.91** |
| **DQ640908** |  | **14010.99** | **4.87** | **131** | **17085/16960** | **33.53** | **-0.032** | **91.60** |  | **DQ663539** |  | **14159.31** | **5.22** | **131** | **17085/16960** | **27.25** | **-0.068** | **82.67** |
| **DQ117911** | **Manzanilla Sevilla** | **14515.46** | **5.06** | **134** | **17085/16960** | **27.96** | **-0.214** | **78.58** |  | **DQ663540** |  | **14129.28** | **5.22** | **131** | **17085/16960** | **27.25** | **-0.049** | **83.44** |
| **DQ138324** |  | **14475.45** | **5.21** | **134** | **17085/16960** | **26.52** | **-0.205** | **75.67** |  | **DQ663541** |  | **14103.20** | **5.22** | **131** | **17085/16960** | **27.03** | **-0.085** | **79.69** |
| **DQ138325** |  | **14427.39** | **5.21** | **134** | **17085/16960** | **26.52** | **-0.172** | **79.33** |  | **DQ663542** |  | **14118.25** | **5.22** | **131** | **17085/16960** | **27.68** | **-0.044** | **81.91** |
| **DQ138326** |  | **14484.49** | **5.39** | **134** | **17085/16960** | **26.09** | **-0.199** | **79.33** |  | **X73279** | ***Zea mays*** | **14147.20** | **4.94** | **131** | **17085/16960** | **32.91** | **-0.127** | **81.91** |
| **DQ317573** | **Morrut** | **14384.37** | **5.06** | **134** | **17085/16960** | **27.96** | **-0.167** | **82.24** |  | **X73280** |  | **14810.97** | **5.09** | **137** | **17085/16960** | **35.45** | **-0.166** | **74.82** |
| **DQ317574** |  | **14348.26** | **5.06** | **134** | **17085/16960** | **28.59** | **-0.216** | **76.42** |  | **X73281** |  | **14237.36** | **4.91** | **131** | **17085/16960** | **31.03** | **-0.047** | **84.81** |
| **DQ317575** |  | **14447.40** | **5.21** | **134** | **17085/16960** | **25.74** | **-0.201** | **75.67** |  | **DQ663559** |  | **14236.42** | **5.24** | **131** | **17085/16960** | **31.14** | **-0.050** | **84.81** |
| **DQ317576** |  | **14418.38** | **5.06** | **134** | **17085/16960** | **27.96** | **-0.175** | **79.33** |  | **DQ663560** |  | **14249.36** | **4.91** | **131** | **17085/16960** | **29.63** | **-0.099** | **76.64** |
| **DQ317580** | **Picual** | **14425.44** | **5.21** | **134** | **17085/16960** | **26.52** | **-0.154** | **81.49** |  | **DQ663561** |  | **14149.23** | **5.22** | **131** | **17085/16960** | **31.04** | **-0.133** | **78.93** |
| **DQ317581** |  | **14413.38** | **5.21** | **134** | **17085/16960** | **26.52** | **-0.193** | **78.58** |  | **DQ663562** |  | **14263.38** | **4.91** | **131** | **17085/16960** | **30.28** | **-0.097** | **77.40** |
| **DQ317582** |  | **14413.38** | **5.21** | **134** | **17085/16960** | **26.52** | **-0.193** | **78.58** |  | **DQ663563** |  | **14245.35** | **4.91** | **131** | **17085/16960** | **30.28** | **-0.082** | **80.38** |
| **DQ640904** |  | **14038.17** | **5.04** | **131** | **17085/16960** | **30.73** | **0.015** | **83.44** |  | **DQ663564** |  | **14189.26** | **4.91** | **131** | **17085/16960** | **34.70** | **-0127** | **76.64** |
| **DQ663553** |  | **14159.31** | **5.22** | **131** | **17085/16960** | **27.25** | **-0.068** | **82.67** |  | **DQ663565** |  | **14163.27** | **5.24** | **130** | **17085/16960** | **28.30** | **-0.120** | **75.00** |
