# Supplementary material for: Characterization of Profilin Polymorphism in Pollen with a Focus on Multifunctionality
Source: PLoS One. 2012 Feb 14;7(2):e30878. doi: 10.1371/journal.pone.0030878 (PMC3279341; doi:10.1371/journal.pone.0030878)
Supplement: Table S2 — Analysis of the polymorphism affecting posttranslational modification motifs in the profilin sequences. The most representative and important posttranslational modifications were examined for the profilin amino acid sequences. These motifs included: N-myristoylation, amidation, phosphorylation by MAP kinase, N-glycosilation and targeting signal for microsomal bodies. (DOC) [file pone.0030878.s002.doc]

**Table S2. Analysis of the polymorphism affecting posttranslational modification motifs in the profilin sequences.**

| **GenBank Accession N°** | ***Olea europaea* L.**  **Cultivar** | **N-Myristoylation** | **Amidation** | **MAPKase Phosphorilation**  **(PVTP)/(IQGEAGAVIRGKKGSGGITIK)** | **N-Glycosilation** | **Microbodies Targeting Signal** |  | **GenBank Accession N°** | ***Olea europaea* L.**  **Cultivar** | **N-Myristoylation** | **Amidation** | **MAPKase Phosphorilation**  **(PVTP)/(IQGEAGAVIRGKKGSGGITIK)** | **N-Glycosilation** | **Microbodies Targeting Signal** |
| --- | --- | --- | --- | --- | --- | --- | --- | --- | --- | --- | --- | --- | --- | --- |
| **Y12425** | **-** | **33-38 GSvwAQ**  **67-72 GLhlGG** | **87-90**  **rGKK** | **-/-** | - | **-** |  | **DQ663555** | **Picual** | **30-35 GTvwAQ**  **64-69 GMfvAT** | **87-90**  **rGKK** | **PMTP/IQGEPGAVIRGKKGAGGITIK** | **-** | **-** |
| **Y12429** | **-** |  |  |  |  |  |  | **DQ663556** |  |  |  |  |  |  |
| **Y12430** | **-** |  |  |  |  |  |  | **DQ663557** |  | **30-35 GAvwAQ** |  | **PMTP/IQGEPGAVIRGKKGSGGITVK** |  |  |
| **DQ138355** | **Acebuche** |  | **87-90**  **rGKK** | **-/-** | - | **-** |  | **DQ663558** |  | **30-35 GTvwAQ**  **64-69 GMfvAT** |  | **PMTP/IAGEPGAVIRGKKGSGGITIK** |  | **128-130 NRA** |
| **DQ138356** |  |  |  |  |  |  |  | **DQ117907** | **Picudo** | **33-38 GSvwAQ**  **67-72 GLhlGG** | **87-90**  **rGKK** | **-/-** | **-** | **-** |
| **DQ138357** |  |  |  |  |  |  |  | **DQ117909** |  |  |  |  |  |  |
| **DQ138327** | **Arbequina** | **33-38 GSvwAQ**  **67-72 GLhlGG** | **87-90**  **rGKK** | **-/-** | - | **-** |  | **DQ117908** |  | **30-35 GQdgSV**  **33-38 GSvwAQ**  **67-72 GLhlGG** |  |  |  |  |
| **DQ138328** |  |  |  |  |  |  |  | **DQ117910** |  |  |  |  |  |  |
| **DQ138329** |  |  |  |  |  |  |  | **DQ138348** | **Sevillenca** | **33-38 GSvwAQ**  **67-72 GLhlGG** | **87-90**  **rGKK** | **-/-** | **-** | **-** |
| **DQ138330** |  |  |  |  |  |  |  | **DQ138349** |  |  |  |  |  |  |
| **DQ317563** | **Bella de España** | **33-38 GSvwAQ**  **67-72 GLhlGG** | **87-90**  **rGKK** | **-/IQGEAGAVVRGKKGSGGITIK** | - | **-** |  | **DQ138350** |  |  |  |  |  |  |
| **DQ317564** |  |  |  | **-/VQGEAGAVIRGKKGSGGITIK** |  |  |  | **DQ317577** | **Sourani** | **33-38 GSvwAQ**  **67-72 GLhlGG** | **87-90**  **rGKK** | **-/-** | **-** | **-** |
| **DQ640909** |  | **30-35 GTvwAQ**  **64-69 GMfvAG** |  | **PMTP/IQGEPGAVIRGKKGAGGITIK** |  |  |  | **DQ317578** |  |  |  |  |  |  |
| **DQ640910** |  | **17-22 GQhlTA**  **30-35 GSvwAQ**  **64-69 GLhlGG** |  | **PLTP/IQGEPGAVIRGKKGAGGITVK** |  |  |  | **DQ317579** |  |  |  |  |  |  |
| **DQ138335** | **Blanqueta** | **33-38 GSvwAQ**  **67-72 GLhlGG** | **87-90**  **rGKK** | **-/-** | - | **-** |  | **DQ640905** |  | **17-22 GQhlTA**  **30-35 GSvwAQ**  **64-69 GLhlGG** | **84-87**  **rGKK** | **PLTP/IRGEPGAVIRGKKGAGGITVK** |  |  |
| **DQ138336** |  | **30-35 GQdgSV**  **33-38 GSvwAQ**  **67-72 GLhlGG** |  | **-/IAGEPGAVIRGKKGAGGITIK** |  |  |  | **DQ117902** | **Verdial Huevar** | **33-38 GSvwAQ**  **67-72 GLhlGG** | **87-90**  **rGKK** | **-/-** | **-** | **-** |
| **DQ138337** |  | **33-38 GSvwAQ**  **67-72 GLhlGG** |  | **-/-** | **51-54 NGTM** |  |  | **DQ117903** |  |  |  |  |  |  |
| **DQ138338** |  |  |  |  | - |  |  | **DQ117905** |  |  |  |  |  |  |
| **DQ138331** | **Cornicabra** | **67-72 GLhlGG** | **87-90**  **rGKK** | **-/-** | - | **-** |  | **DQ117904** |  |  |  |  |  |  |
| **DQ138332** |  | **33-38 GSvwAQ**  **67-72 GLhlGG** |  |  |  |  |  | **DQ117906** |  |  |  |  |  |  |
| **DQ138333** |  |  |  |  |  |  |  | **DQ138359** | **Verdial Málaga** | **33-38 GSvwAQ**  **67-72 GLhlGG** | **87-90**  **rGKK** | **-/-** | **-** | **-** |
| **DQ138334** |  |  |  |  |  |  |  | **DQ138358** |  |  |  | **-/IQGEAGAVTRGKKGTGGITIK** |  |  |
| **DQ138342** | **Empeltre** | **33-38 GSvwAQ**  **67-72 GLhlGG** | **87-90**  **rGKK** | **-/-** | - | **-** |  | **DQ138360** |  |  |  | **-/-** |  |  |
| **DQ138343** |  |  |  |  |  |  |  | **DQ138361** |  |  |  |  |  |  |
| **DQ138344** |  |  |  |  |  |  |  | **DQ138351** | **Villalonga** | **33-38 GSvwAQ**  **67-72 GLhlGG** | **87-90**  **rGKK** | **SVTP/-** | **-** | **-** |
| **DQ317565** | **Farga** | **33-38 GSvwAQ**  **67-72 GLhlGG** | **87-90**  **rGKK** | **-/-** | - | **-** |  | **DQ138353** |  |  |  | **PVAP/-** |  |  |
| **DQ317566** |  |  |  |  |  |  |  | **DQ138352** |  |  |  |  |  |  |
| **DQ317567** |  |  |  |  |  |  |  | **DQ138354** |  |  |  |  |  |  |
| **DQ317568** | **Frantoio** | **33-38 GSvwAQ**  **67-72 GLhlGG** | **87-90**  **rGKK** | **-/-** | - | **-** |  | **DQ640907** |  | **30-35 GTvwAQ**  **64-69 GMfvAT** | **84-87**  **rGKK** | **PMTP/IAGEPGAVIRGKKGSGGITIK** |  |  |
| **DQ317569** |  |  |  |  |  |  |  |  |  |  |  |  |  |  |
| **DQ317570** | **Galega** | **33-38 GSvwAQ**  **67-72 GLhlGG**  **93-98 GGitTK** | **87-90**  **rGKK** | **-/-** | - | **-** |  |  |  |  |  |  |  |  |
| **DQ061979** | **Hojiblanca** | **33-38 GSvwAQ**  **67-72 GLhlGG** | **87-90**  **rGKK** | **-/-** | - | **-** |  | **GenBank Accession N°** | **Specie** | **N-Myristoylation** | **Amidation** | **MAPKase Phosphorilation**  **(PVTP)/(IQGEAGAVIRGKKGSGGITIK)** | **N-Glycosilation** | **Microbodies Targeting Signal** |
| **DQ061980** |  |  |  |  |  |  |  |  |  |  |  |  |  |  |
| **DQ061981** |  |  |  |  |  |  |  | **M65179** | ***Betula pendula*** | **(17/19)-24 GQqlAA**  **32-37 GSvwAQ**  **66-71 GLhlGG** | **86-89**  **rGKK** | **-/-** | **-** | **-** |
| **DQ061982** |  | **30-35 GQdgSV**  **33-38 GSvwAQ**  **67-72 GLhlGG** |  |  |  |  |  | **DQ650633** |  |  |  |  |  |  |
| **DQ138345** | **Leccino** | **33-38 GSvwAQ**  **67-72 GLhlGG** | **87-90**  **rGKK** | **-/-** | - | **-** |  | **DQ663544** | ***Corylus avellana*** | **19-24 GQqlAA**  **32-37 GSvwAQ**  **66-71 GLhlGG** | **86-89**  **rGKK** | **PVTP/-** | **-** | **-** |
| **DQ138346** |  |  |  |  |  |  |  | **DQ663546** |  |  |  |  |  |  |
| **DQ138347** |  |  |  |  |  |  |  | **DQ663547** |  |  |  | **PMTP/IQGEPGVVIRGKKGAGGITIK** |  |  |
| **DQ317571** | **Lechín de Granada** | **33-38 GSvwAQ**  **67-72 GLhlGG** | **87-90**  **rGKK** | **-/-** | - | **-** |  | **DQ663548** |  |  |  | **PVTP/-** |  |  |
| **DQ317572** |  |  |  |  |  |  |  | **DQ663549** |  |  |  |  |  |  |
| **DQ640906** |  | **17-22 GLhlAS**  **30-35GTvwAQ**  **64-69 GMfvAG** |  | **PMTP/IQGEPGAVIRGKKGAGGITIK** |  |  |  | **DQ663550** |  |  |  |  |  |  |
| **DQ028766** | **Lechín de Sevilla** | **33-38 GSvwAQ**  **67-72 GLhlGG** | **87-90**  **rGKK** | **-/-** | - | **-** |  | **DQ663551** |  | **30-35 GSvwAQ**  **64-69 GLhlGG** |  |  |  |  |
| **DQ061976** |  |  |  |  |  |  |  | **DQ663552** |  |  |  |  |  |  |
| **DQ061977** |  |  |  |  |  |  |  | **DQ663543** |  | **19-24 GQqlAA**  **32-37 GSvwAQ**  **66-71 GLhlGG** | **86-89**  **rGKK** |  |  |  |
| **DQ061978** |  |  |  |  |  |  |  | **DQ663545** |  |  |  | **PLTP/IQGESGAVIRGKKGAGGITVK** |  |  |
| **DQ138339** | **Loaime** | **33-38 GSvwAQ**  **67-72 GLhlGG** | **87-90**  **rGKK** | **-/-** | - | **-** |  |  |  |  |  |  |  |  |
| **DQ138340** |  |  |  |  |  |  |  | **X77583** | ***Phleum pratense*** | **30-35 GTvwAQ**  **64-69 GMfvAG** | **84-87**  **rGKK** | **PMTP/IQGEPGRVIRGKKGAGGITIK** | **-** | **-** |
| **DQ138341** |  |  |  |  |  |  |  | **Y09456** |  |  |  | **PMTP/IQGEPGAVIRGKKGAGGITIK** |  |  |
| **DQ640903** |  | **30-35 GQdgSV**  **33-38 GSvwAQ**  **67-72 GLhlGG** |  | **-/IQGEPGAVIRGKKGSGGITIK** |  |  |  | **Y09457** |  |  |  |  |  |  |
| **DQ138362** | **Lucio** | **33-38 GSvwAQ**  **67-72 GLhlGG** | **87-90**  **rGKK** | **-/-** | - | **-** |  | **Y09458** |  |  |  |  |  |  |
| **DQ138363** |  | **30-35 GQdgSV**  **33-38 GSvwAQ**  **67-72 GLhlGG** |  |  |  |  |  | **DQ663535** |  | **30-35 GTvwAQ**  **64-69 GMfvAT** |  |  |  |  |
| **DQ138365** |  | **33-38 GSvwAQ**  **67-72 GLhlGG** |  |  |  |  |  | **DQ663538** |  |  |  |  |  |  |
| **DQ138364** |  |  |  |  |  |  |  | **DQ663539** |  |  |  |  |  |  |
| **DQ640908** |  | **17-22 GQhlTA**  **33-38 GSvwAQ**  **67-72 GLhlGG** |  | **PLTP/IQGEPGAVIRGKKGAGGITVK** |  |  |  | **DQ663542** |  |  |  |  |  |  |
| **DQ117911** | **Manzanilla Sevilla** | **33-38 GSvwAQ**  **67-72 GLhlGG** | **87-90**  **rGKK** | **-/-** | - | **-** |  | **DQ663536** |  | **30-35 GTvwAQ**  **64-69 GMfvAA** |  |  |  |  |
| **DQ138324** |  |  |  |  |  |  |  | **DQ663537** |  |  |  |  |  |  |
| **DQ138325** |  |  |  |  |  |  |  | **DQ663540** |  |  |  |  |  |  |
| **DQ138326** |  |  |  |  |  |  |  | **DQ663541** |  |  |  |  |  |  |
| **DQ317573** | **Morrut** | **30-35 GQdgSV**  **33-38 GSvwAQ**  **67-72 GLhlGG** | **87-90**  **rGKK** | **-/-** | **-** | **-** |  | **X73279** | ***Zea mays*** | **30-35 GAtwAQ**  **64-69 GLilGG** | **84-87**  **rGKK** | **PMTP/IQGEPGAVIRGKKGSGGITVK** | **-** | **-** |
| **DQ317574** |  | **30-35 GQdgSV**  **33-38 GSvwAQ**  **67-72 GLhlGG**  **93-98 GGitSK** |  | **-/IQGEAGAVIRGKKGSGGITSK** |  |  |  | **DQ663564** |  |  |  |  |  |  |
| **DQ317575** |  | **33-38 GSvwAQ**  **67-72 GLhfGG** |  | **-/-** |  |  |  | **X73280** |  | **30-35 GAvwAQ** |  |  |  |  |
| **DQ317576** |  | **30-35 GQdgSV**  **33-38 GSvwAQ**  **67-72 GLhlGG** |  |  |  |  |  | **X73281** |  | **36-41 GAawAQ** |  |  |  |  |
| **DQ317580** | **Picual** | **33-38 GSvwAQ**  **67-72 GLhlGG** | **87-90**  **rGKK** | **-/-** | **-** | **-** |  | **DQ663559** |  |  |  |  |  |  |
| **DQ317581** |  |  |  |  |  |  |  | **DQ663560** |  | **30-35 GAvwAQ** |  |  |  |  |
| **DQ317582** |  |  |  |  |  |  |  | **DQ663561** |  |  |  |  |  |  |
| **DQ640904** |  | **30-35 GTvwAQ**  **64-69 GMfvAT** |  | **PMTP/IQGEPGAVIRGKKGAGGITIK** |  |  |  | **DQ663562** |  |  |  |  |  |  |
| **DQ663553** |  |  |  |  |  |  |  | **DQ663563** |  |  |  |  |  |  |
| **DQ663554** |  |  |  |  |  |  |  | **DQ663565** |  |  |  |  |  | **128-130 NRA** |
