# Supplementary material for: Characterization of Profilin Polymorphism in Pollen with a Focus on Multifunctionality
Source: PLoS One. 2012 Feb 14;7(2):e30878. doi: 10.1371/journal.pone.0030878 (PMC3279341; doi:10.1371/journal.pone.0030878)
Supplement: Table S3 — The polymorphism of putative phosphorylation residues. Serine, threonine and tyrosine residues of the profilin sequences were analyzed. Multiple combinations of residues for susceptible phosphorylation were found among profilin sequences. (DOC) [file pone.0030878.s003.doc]

**Table S3. The polymorphism of putative phosphorylation residues.**

| **GenBank**  **Accession N°** | ***Olea europaea* L. Cultivar** | **Ser (S)** | **Thr (T)** | **Tyr (Y)** |  | **GenBank**  **Accession N°** | ***Olea europaea* L. Cultivar** | **Ser (S)** | **Thr (T)** | **Tyr (Y)** |
| --- | --- | --- | --- | --- | --- | --- | --- | --- | --- | --- |
| **Y12425** | **-** | **- S92 -** | **- T96 -** | **- Y6 - / - Y75 -** |  | **DQ663555** | **Picual** | **- S92 -** | **- T96 -** | **- Y6 - / - Y75 -** |
| **Y12429** | **-** |  |  |  |  | **DQ663556** |  |  |  |  |
| **Y12430** | **-** |  | **- T5 - / - T96 -** |  |  | **DQ663557** |  |  |  | **- Y6 - / - Y75 - / - Y109 -** |
| **DQ138355** | **Acebuche** | **- S92 -** | **- T5 - / - T96 -** | **- Y6 - / - Y75 - / - Y109 -** |  | **DQ663558** |  |  |  |  |
| **DQ138356** |  |  | **- T5 - / - T93 - / - T111 -** | **- Y6 - / - Y72 -** |  | **DQ117907** | **Picudo** | **- S92 -** | **- T96 -** | **- Y6 - / - Y75 -** |
| **DQ138357** |  | **-** | **- T93 - / - T111 -** | **- Y6 -** |  | **DQ117908** |  |  |  |  |
| **DQ138327** | **Arbequina** | **- S92 -** | **- T5 - / - T96 -** | **- Y6 - / - Y75 -** |  | **DQ117910** |  |  |  |  |
| **DQ138328** |  |  | **- T96 -** | **- Y6 - / - Y75 - / - Y109 -** |  | **DQ117909** |  |  | **- T5 - / - T96 -** | **- Y6 - / - Y75 - / - Y109** |
| **DQ138329** |  |  |  | **- Y6 - / - Y75 -** |  | **DQ138348** | **Sevillenca** | **-** | **- T93 - / - T111 -** | **- Y6 - / - Y72 -** |
| **DQ138330** |  |  |  |  |  | **DQ138349** |  | **- S92 -** | **- T96 -** | **- Y6 - / - Y75 -** |
| **DQ317563** | **Bella de España** | **- S92 -** | **- T96 -** | **- Y6 - / - Y75 -** |  | **DQ138350** |  |  |  | **- Y75 -** |
| **DQ640909** |  |  |  |  |  | **DQ317577** | **Sourani** | **- S92 -** | **- T96 -** | **- Y6 - / - Y75 -** |
| **DQ640910** |  |  | **- T5 - / - T95 -** | **- Y6 - / - Y75 - / - Y109 -** |  | **DQ317578** |  |  |  | **- Y6 - / - Y75 - / - Y109** |
| **DQ317564** |  |  | **- T96 -** | **- Y6 - / - Y128 -** |  | **DQ640905** |  |  |  |  |
| **DQ138335** | **Blanqueta** | **- S92 -** | **- T96 -** | **- Y6 - / - Y75 -** |  | **DQ317579** |  |  | **- T5 - / - T93 - / - T111 -** | **- Y6 - / - Y72 -** |
| **DQ138336** |  |  |  |  |  | **DQ117903** | **Verdial Huevar** | **- S92 -** | **- T96 -** | **- Y6 - / - Y75 - / - Y109 -** |
| **DQ138338** |  |  |  |  |  | **DQ117902** |  |  |  | **- Y6 - / - Y75 -** |
| **DQ138337** |  |  |  | **- Y6 -** |  | **DQ117904** |  |  |  |  |
| **DQ138331** | **Cornicabra** | **-** | **- T5 - / - T55 - / - T96 -** | **- Y6 -** |  | **DQ117905** |  |  | **- T5 - / - T96 -** |  |
| **DQ138332** |  | **- S92 -** | **- T96 -** | **- Y6 - / - Y75 -** |  | **DQ117906** |  |  |  |  |
| **DQ138333** |  |  |  |  |  | **DQ138358** | **Verdial Málaga** | **- S92 -** | **- T96 -** | - **Y6 - / - Y75 -** |
| **DQ138334** |  |  | **- T5 - / - T96 -** |  |  | **DQ138360** |  |  |  |  |
| **DQ138342** | **Empeltre** | **- S92 -** | **- T96 - / - T97 -** | **- Y6 - / - Y75 -** |  | **DQ138361** |  |  | **- T5 -/- T96 -** | **- Y75 -** |
| **DQ138343** |  |  | **- T96 -** | **- Y6 - / - Y75 - / - Y109 -** |  | **DQ138359** |  |  | **- T96 -** |  |
| **DQ138344** |  |  | **- T5 - / - T96 -** |  |  | **DQ138351** | **Villalonga** | **- S92 -** | **- T96 -** | **- Y6 - / - Y75 - / - Y109 -** |
| **DQ317565** | **Farga** | **- S42 - / - S92 -** | **- T5 - / - T96 -** | **- Y6 - / - Y75 - / - Y109 -** |  | **DQ138352** |  |  | **- T55 - / - T96 -** |  |
| **DQ317567** |  | **- S92 -** |  | **- Y6 - / - Y75 -** |  | **DQ138353** |  |  |  |  |
| **DQ317566** |  |  | **- T96 -** |  |  | **DQ640907** |  |  |  |  |
| **DQ317568** | **Frantoio** | **-** | **- T5 - / - T93 - / - T111-** | **- Y6 -** |  | **DQ138354** |  | **-** | **- T93 - / - T111 -** | **- Y6 - / - Y72 -** |
| **DQ317569** |  | **- S92 - / - S97 -** | **- T96 -** | **- Y6 - / - Y75 -** |  |  |  |  |  |  |
| **DQ317570** | **Galega** | **- S92 -** | **- T96 -** | **- Y6 - / - Y75 -** |  |  |  |  |  |  |
| **DQ061979** | **Hojiblanca** | **- S92 -** | **-** | **- Y6 - / - Y75 -** |  |  |  |  |  |  |
| **DQ061980** |  |  | **- T96 -** |  |  | **GenBank**  **Accession N°** | **Specie** | **Ser (S)** | **Thr (T)** | **Tyr (Y)** |
| **DQ061981** |  |  | **- T5 - / - T96 -** |  |  |  |  |  |  |  |
| **DQ061982** |  |  | **- T96 -** | **- Y6 - / - Y75 - / - Y109 -** |  | **M65179** | ***Betula pendula*** | **-** | **- T93- / - T111 -** | **- Y6 -** |
| **DQ138345** | **Leccino** | **-- S58 - / - S92** | **- T96 -** | **- Y6 - / - Y75 - / - Y109 -** |  | **DQ650633** |  |  |  |  |
| **DQ138346** |  | **- S92 -** | **- T5 - / - T73 - / -T96 -** |  |  | **DQ663543** | ***Corylus avellana*** | **- S91 -** | **- T5 - / - T95 -** | **- Y6 - / - Y74 -** |
| **DQ138347** |  |  | **-T96-** | **- Y6 - / - Y75 -** |  | **DQ663544** |  |  |  |  |
| **DQ317571** | **Lechín de Granada** | **- S92 -** | **- T96 -** | **- Y6 - / - Y75 - / - Y109 -** |  | **DQ663545** |  | **- S89 -** | **- T5 - / - T93 - / - T111 -** | **- Y6 - / - Y72 -** |
| **DQ317572** |  |  |  |  |  | **DQ663546** |  |  |  |  |
| **DQ640906** |  |  | **T5 - / - T96 -** |  |  | **DQ663548** |  |  | **- T37 - / - T44 - / - T93 - / - T111 -** |  |
| **DQ028766** | **Lechín de Sevilla** | **- S5 - / - S92 -** | **- T96 -** | **- Y6 - / - Y75 -** |  | **DQ663547** |  |  | **- T93 - / - T111 -** |  |
| **DQ061976** |  | **- S92 -** |  |  |  | **DQ663549** |  |  |  |  |
| **DQ061977** |  |  |  |  |  | **DQ663550** |  |  |  |  |
| **DQ061978** |  |  |  |  |  | **DQ663551** |  | **- S91 -** | **- T5 - / - T95 -** | **- Y6 - / - Y74 -** |
| **DQ138339** | **Loaime** | **- S92 -** | **- T96 -** | **- Y6 - / - Y75 -** |  | **DQ663552** |  |  |  |  |
| **DQ138340** |  |  |  |  |  | **X77583** | ***Phleum pratense*** | **- S98 -** | **- T93 - / - T111 -** | **- Y6 -** |
| **DQ138341** |  |  |  |  |  | **Y09456** |  | **- S91 -** | **- T95 -** | **- Y6 - / - Y74 -** |
| **DQ640903** |  | **-** | **T5 - / - T96 - / - T111 -** | **- Y6 - / - Y72 -** |  | **Y09457** |  |  |  |  |
| **DQ138362** | **Lucio** | **- S92 -** | **- T96 -** | **- Y6 - / - Y75 -** |  | **DQ663540** |  |  |  |  |
| **DQ138363** |  |  |  |  |  | **DQ663542** |  |  |  |  |
| **DQ138365** |  |  |  |  |  | **Y09458** |  | **-** | **- T93 - / - T111 -** | **- Y6 -** |
| **DQ138364** |  |  |  |  |  | **DQ663535** |  |  |  |  |
| **DQ640908** |  | **-** | **- T5 - / - T96 -** |  |  | **DQ663536** |  |  | **- T5 - / - T93 - / - T111 -** |  |
| **DQ117911** | **Manzanilla Sevilla** | **- S92 -** | **- T96 -** | **- Y6 - / - Y75 - / - Y109 -** |  | **DQ663537** |  |  |  |  |
| **DQ138324** |  |  |  | **- Y6 - / - Y75 -** |  | **DQ663538** |  |  |  |  |
| **DQ138325** |  |  |  |  |  | **DQ663539** |  |  |  |  |
| **DQ138326** |  |  |  |  |  | **DQ663541** |  |  |  |  |
| **DQ317573** | **Morrut** | **- S92 -** | **- T96 -** | **- Y6 - / - Y75 - / - Y109 -** |  | **X73279** | ***Zea mays*** | **-** | **- T93 - / - T111 -** | **- Y6 -/ - Y72 -** |
| **DQ317574** |  |  |  |  |  | **X73280** |  |  |  | **- Y6 -** |
| **DQ317575** |  |  | **- T5 - / - T96 -** |  |  | **X73281** |  |  |  |  |
| **DQ317576** |  | **-** | **- T93 - / - T111 -** | **- Y6 -** |  | **DQ663560** |  | **- S8 - / - S95 -** | **- T43 - / - T50 - / - T99 - / - T117-** | **- Y6 - / - Y74 -** |
| **DQ317580** | **Picual** | **-** | **- T93 - / - T111 -** | **- Y6 -** |  | **DQ663559** |  | **- S89 -** | **- T5 - / - T37 - / - T93 - / - T111 -** | **- Y6 - / - Y72 -** |
| **DQ317581** |  |  |  |  |  | **DQ663561** |  |  | **- T5 - / - T93 - / - T111 -** |  |
| **DQ317582** |  |  |  |  |  | **DQ663562** |  | **-** |  |  |
| **DQ640904** |  |  |  |  |  | **DQ663563** |  | **- S92 -** | **- T96 -** | **- Y6 -** |
| **DQ663553** |  | **- S92 -** |  | **- Y6 - / - Y72 -** |  | **DQ663565** |  | **-** | **- T93 - / - T111 -** | **- Y6 -/ - Y72 -** |
| **DQ663554** |  |  | **- T96 -** | **- Y6 - / - Y75 -** |  | **DQ663564** |  |  |  | **- Y6 -** |
